# From Floral By‐Product to Bioactive Resource: Phytochemical Profiling and Antioxidant Potential of *Crocus sativus* Stamens

**DOI:** 10.1002/fsn3.71282

**Published:** 2025-12-13

**Authors:** Samira Mamri, Sanae Baddaoui, Dilaycan Çam, Cansel Çakir, Mohammed Roubi, Raffaele Conte, Yusuf Sıcak, Mehmet Öztürk, Mohammed Choukri, Musaab Dauelbait, Gehan M. Elossaily, Yousef A. Bin Jardan, Abdel‐Rhman Z. Gaafar, Abdeslam Asehraou, Ennouamane Saalaoui

**Affiliations:** ^1^ Laboratory of Bioresources, Biotechnology, Ethnopharmacology and Health, Department of Biology, Faculty of Sciences Mohammed First University Oujda Morocco; ^2^ Department of Chemistry, Faculty of Sciences Muğla Sıtkı Koçman University Muğla Türkiye; ^3^ Research Institute on Terrestrial Ecosystems (IRET) National Research Council of Italy (CNR) Naples Italy; ^4^ Department of Medicinal and Aromatic Plants, Köyceğiz Vocational School Mugla Sitki Kocman University Muğla Türkiye; ^5^ Faculty of Medicine and Pharmacy Mohammed First University Oujda Morocco; ^6^ Maternal‐Child and Mental Health Research Laboratory, Faculty of Medicine and Pharmacy Mohammed First University Oujda Morocco; ^7^ University of Bahr el Ghazal Wau South Sudan; ^8^ Department of Basic Medical Sciences, College of Medicine AlMaarefa University Riyadh Saudi Arabia; ^9^ Research Center, Deanship of Scientific Research and Post‐Graduate Studies AlMaarefa University Riyadh Saudi Arabia; ^10^ Department of Pharmaceutics, College of Pharmacy King Saud University Riyadh Saudi Arabia; ^11^ Department of Botany and Microbiology, College of Science King Saud University Riyadh Saudi Arabia

**Keywords:** *Crocus sativus*, floral by‐products, mineral composition, stamens valorization, UHPLC–MS/MS

## Abstract

The production of saffron ( *Crocus sativus* L.) generates large quantities of floral by‐products, particularly stamens, which are often discarded despite their potential as sources of bioactive compounds. This study aimed to provide, for the first time, a comprehensive and comparative characterization of *C. sativus* stamen extracts and fractions using a multianalytical approach (UHPLC–MS/MS, GC–MS, ICP‐OES, and spectrophotometry), in order to reveal their nutritional, phytochemical, and antioxidant potential. Elemental analyses revealed high levels of essential macro‐ and micronutrients such as potassium, phosphorus, calcium, and iron. Spectrophotometric and chromatographic assays quantified sugars and proteins, while UHPLC–MS/MS and HPLC‐DAD identified key phenolic acids and flavonoids, including chlorogenic acid, rutin, quercetin, and crocin. GC–MS profiling of the petroleum ether fraction showed palmitic and oleic acids as major components. The hydrolyzed ethyl acetate fraction displayed the highest antioxidant activity across DPPH, ABTS, β‐carotene bleaching, and metal‐chelating assays, likely due to the release of aglycone phenolics and synergistic effects. This work introduces an original integrative analysis of saffron stamens, highlighting them as an underexplored and sustainable source of bioactive nutrients and antioxidants with promising applications in the food, nutraceutical, and cosmetic industries.

## Graphical abstract

This study highlights the phytochemical richness and nutritional value of *Crocus sativus* stamens, characterized through UHPLC–MS/MS and GC–MS analyses. The extracts and fractions showed significant antioxidant potential, supported by multiple in vitro assays. Overall, the findings demonstrate the potential of saffron stamens as a valuable natural source of bioactive and nutritionally relevant compounds.

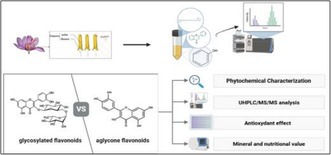

## 1 Introduction

Plant‐derived natural antioxidants have attracted growing scientific interest due to their potential to counteract oxidative stress, a key factor in the development of various diseases such as cardiovascular disorders, neurodegenerative conditions, and certain types of cancer (Lobo et al. 2010). Among these bioactive compounds, phenolics and flavonoids are especially recognized for their potent radical scavenging properties, metal‐chelating capacities, and ability to modulate oxidative enzyme activity (Gulcin and Alwasel 2022; Mamri, Daoudi, Marghich, et al. 2022; El‐Lateef et al. 2023).

*Crocus sativus* , commonly known as saffron, is a short‐lived perennial species of the Iridaceae family (Mykhailenko et al. 2019; Mamri, Daoudi, Marghich, et al. 2022). It is primarily cultivated for its dried stigmas, which are among the most valuable spices worldwide. According to the Moroccan Ministry of Agriculture (2020), Morocco ranks as the fourth‐largest saffron producer worldwide and the leading producer in Africa. By 2019, the cultivated area had expanded to 1860 ha, yielding 6.5 tons of saffron and engaging more than 4300 farmers. This growth significantly boosted revenues, which rose to 139 million MAD in 2018—nearly nine times higher than the 16 million MAD recorded in 2008.

Despite Safron's economic importance, its production generates large volumes of by‐products, including tepals, stamens, leaves, and corms, which constitute over 90% of the harvested plant biomass. These by‐products are generally discarded due to their low commercial value (Serrano‐Díaz et al. 2012; Ahmadi Shadmehri et al. 2019). Specifically, for each kilogram of dried saffron stigmas, approximately 63 kg of floral biowaste are produced comprising about 53 kg of tepals, 9 kg of stamens, and 1 kg of styles along with 1.500 kg of leaves, and 100 kg each of spathes and corms (Cardone et al. 2020). This substantial biomass remains underutilized, both in terms of recovery and potential applications in the food and health sectors. Consequently, environmentally friendly strategies are necessary to explore and develop valuable uses for saffron floral residues. Their valorization could significantly enhance the sustainability of *Crocus sativus* cultivation (Moratalla‐López et al. 2019; Cardone et al. 2020).

Among the less‐exploited parts, stamens have attracted increasing scientific interest due to their content in phenolic acids, flavonoids, and other bioactive compounds, including chlorogenic acid, gallic acid, caffeic acid, and quercetin derivatives (Serrano‐Díaz, Sánchez, Alvarruiz, and Alonso 2013; Babaei et al. 2014; Mamri et al. 2024). Although stamens typically exhibit lower total phenolic content compared to stigmas or tepals, they demonstrate notable antioxidant activity, possibly attributed to highly active minor compounds or synergistic interactions among phytochemicals (Montoro et al. 2012; Serrano‐Díaz, Sánchez, Alvarruiz, and Alonso 2013; Serrano‐Díaz, Sánchez, Martínez‐Tomé, et al. 2013). Recent studies have further emphasized the potential of saffron floral by‐products as promising sources of bioactive molecules, yet these works focused mainly on tepals, leaving the stamen fraction largely unexplored (Jadouali et al. 2019; Khadfy et al. 2023; Ibourki et al. 2024).

The concept of synergistic effect among polyphenols is increasingly recognized as a key factor enhancing the biological effectiveness of plant extracts. Combinations such as (−)‐epigallocatechin gallate with quercetin (Chen et al. 2020), chlorogenic acid with gallic acid (Liu et al. 2021), or caffeic acid with epicatechin gallate (Liu et al. 2022) have shown significantly greater antioxidant activity than individual compounds. These synergistic effects may arise from improved radical stabilization, enhanced bioavailability, or protective interactions between antioxidants (Heim et al. 2002; Shahidi and Zhong 2015).

Such synergistic phenomena may explain the potent antioxidant activity observed in hydrolyzed ethyl acetate fractions of saffron stamens, which are often enriched in aglycone forms of polyphenols with enhanced bioactivity (Alañón et al. 2016; Oracz et al. 2019; Mamri et al. 2025). However, no study to date has simultaneously combined advanced chromatographic, spectroscopic, and elemental techniques to achieve an integrated biochemical characterization of saffron stamens and their bioactive fractions.

Therefore, the present study aimed, for the first time, to conduct a comprehensive and comparative valorization of *Crocus sativus* stamen extracts using a multianalytical strategy (UHPLC–MS/MS, GC–MS, ICP‐OES, and spectrophotometric assays). This integrative approach was designed to elucidate their nutritional composition, phenolic and flavonoid profiles, carotenoids, and volatile compounds, along with their mineral content and antioxidant potential. Various extraction methods, including hydroalcoholic extraction and HCl hydrolysis of ethyl acetate fractions, were compared to identify the most bioactive extracts. The originality of this work lies in the multianalytical integration and the comparative assessment of hydrolyzed versus nonhydrolyzed fractions, offering new insights into the synergistic mechanisms underlying antioxidant activity. The findings provide a scientific basis for the sustainable valorization of saffron stamens as functional ingredients for food, nutraceutical, and cosmetic applications, thereby supporting a circular and eco‐innovative approach within the saffron industry.

## 2 Materials and Methods

### 2.1 Chemicals and Reagents

All chemicals used throughout the experiments were of analytical grade and sourced from recognized suppliers. Methanol, ethanol, ethyl acetate and petroleum ether were purchased from Sigma‐Aldrich (USA). Standard compounds, including rutin, quercetin, caffeic acid, chlorogenic acid, and gallic acid (all ≥ 95% purity), were also obtained from Sigma‐Aldrich and used for calibration in HPLC and other analytical methods. DPPH (2,2‐diphenyl‐1‐picrylhydrazyl), ABTS (2,2′‐azino‐bis (3‐ethylbenzothiazoline‐6‐sulfonic acid)), ferric chloride, and TPTZ (2,4,6‐tripyridyl‐*s*‐triazine), and *β*‐carotene (≥ 90% purity), ferrous sulfate and cupric sulfate were purchased from Sigma‐Aldrich (USA).

### 2.2 Plant Material

*Crocus sativus* L. plants were cultivated in Taliouine, southern Morocco (30°31′54″ N, 7°55′25″ W). The botanical identification of the species was confirmed by Professor Fennane Mohammed. Three voucher samples were deposited at the Herbarium of Mohammed First University in Oujda under the code HUMPOM210. Fresh flowers were collected during the flowering season, and the stamens were carefully separated by hand from the other floral parts. The stamens were then dried at 37°C for 4 h in a ventilated oven. To ensure sample homogeneity, the dried stamens were ground into a fine powder using an electric laboratory mill (IKA MF 10 basic, Germany) and sieved through a 60‐mesh sieve to obtain a fine powder ensuring sample uniformity. The resulting powder was stored at −20°C in airtight containers until further extraction procedures.

### 2.3 Extraction and Fractionation of *Crocus sativus* Stamens

#### 2.3.1 Hydroethanolic Extract (EtOH)

A total of 2 g of the powdered stamens was extracted with 50 mL of an ethanol–water mixture (80:20, v/v) under continuous agitation at 500 rpm. The extraction was performed at room temperature and in the absence of light for 24 h. After filtration through a 0.45 μm membrane, the remaining residue (marc) was subjected to two additional extractions using the same protocol. The pooled extracts were then concentrated under reduced pressure at 40°C with a rotary evaporator and kept at −20°C until further analysis.

#### 2.3.2 Hydromethanolic Extract (MeOH)

The extraction was repeated using a solvent mixture of methanol and water in a ratio of 80:20 (v/v), following the same conditions as described for the hydroethanolic preparation.

#### 2.3.3 Nonhydrolyzed Fractions

**FIGURE 1:**
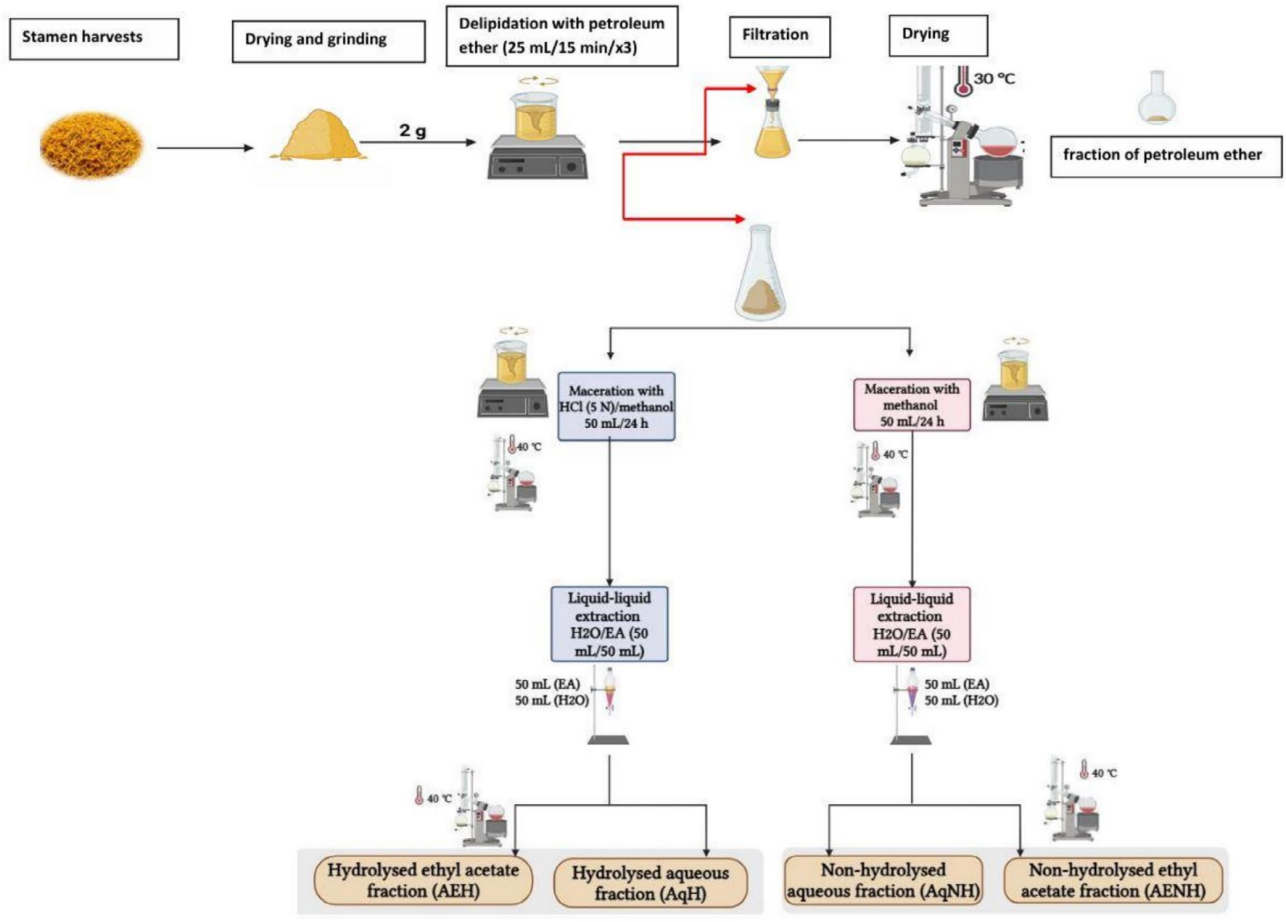
Schematic representation of the sequential extraction and fractionation process applied to *Crocus sativus* stamens.

Two grams of powdered stamens were defatted three times with 25 mL petroleum ether (15 min each, room temperature, orbital shaker). The dried defatted residue was extracted with 50 mL of methanol under constant stirring in the dark for 24 h. After evaporation, the residue was reconstituted in 50 mL of distilled water and subjected to liquid–liquid partitioning with ethyl acetate using a separating funnel. Two fractions were recovered: nonhydrolyzed aqueous fraction (AqNH) and a nonhydrolyzed ethyl acetate fraction (AENH). Both fractions were evaporated and stored at −20°C until further analysis (Figure 1).

#### 2.3.4 HCl‐Hydrolyzed Fractions

Using the same defatting procedure, 2 g of the dried residue were macerated in 50 mL of 5 N hydrochloric acid under continuous stirring in the dark for 2 h. After hydrolysis, the mixture was evaporated to dryness, reconstituted in distilled water, and partitioned with ethyl acetate. This step resulted in two hydrolyzed fractions: hydrolyzed aqueous fraction (AqH) and a hydrolyzed ethyl acetate fraction (AEH). Both fractions were concentrated and stored at −20°C until further analysis (Figure 1).

#### 2.3.5 Petroleum Ether Fraction

The petroleum ether phases from the delipidation step were pooled (225 mL) and evaporated at 30°C. This fraction was analyzed by GC–MS for volatile and fatty acid content (Figure 1).

Extraction yields (%) were calculated on a dry weight basis as the ratio of recovered extract weight to the dry weight of starting material.

### 2.4 Mineral Composition Analysis

#### 2.4.1 Qualitative Analysis by Energy‐Dispersive X‐Ray Fluorescence Spectroscopy (EDXRF)

The stamen powder's elemental profile was analyzed qualitatively using energy‐dispersive X‐ray fluorescence spectroscopy (EDXRF‐7000). Samples were washed with distilled water to remove surface contaminants, dried, and mounted in sample holders for X‐ray excitation. Elemental peaks were identified based on energy emissions (Verma et al. 2022).

#### 2.4.2 Quantitative Analysis by Inductively Coupled Plasma–Optical Emission Spectroscopy (ICP‐OES)

For quantitative mineral analysis, 0.5 g of dried powder was digested in 6 mL HNO_3_ (65%) and 2 mL H_2_O_2_ (30%) using a closed microwave digestion system (CEM Mars 5). After cooling, the solution was filtered and diluted to a final volume of 100 mL. Analyses were performed using an Agilent 7700× ICP‐MS spectrometer. Mineral element concentrations were determined by inductively coupled plasma optical emission spectroscopy (ICP‐OES), following the method described by (Tel‐Cayan et al. 2018). Accuracy was validated using certified reference material (NIST‐CRM‐1203), with RSD < 8%.

### 2.5 Total Sugar Quantification

For total sugar analysis of stamen extracts (MeOH and EtOH), we employed the phenol‐sulfuric acid assay (Lam et al. 2021) with optimized parameters. A 2 mL aliquot of each extract (MeOH and EtOH) with a concentration of 4 mg/mL is mixed with 1 mL of a 5% aqueous phenol solution in a test tube. Next, 5 mL of concentrated sulfuric acid was quickly added. After incubation for 10 min and vigorous shaking for 30 s, the tubes were placed in a water bath at 30°C for 20 min. Absorbance at 480 nm was measured using a microplate reader (BioTek Epoch 2 Microplate Spectrophotometer). Blank samples were prepared by replacing the sugar solution with distilled water. The quantity of total sugars is determined by reference to a calibration curve established from glucose solutions (1 mg/mL), and the results are expressed in mg D‐glucose equivalent (GE) per gram of dry weight of extract (mg GE/g dried extract).

### 2.6 Reducing Sugar Analysis

Reducing sugar content was determined using our modified 3,5‐dinitrosalicylic acid (DNSA) protocol (Krivorotova and Sereikaite 2014). The DNSA reagent was prepared by dissolving 1 g DNSA and 30 g sodium potassium tartrate in 80 mL 0.5 N NaOH at 45°C. After dissolution, the solution was cooled to room temperature and diluted to 100 mL with distilled water. For measurement, 2 mL of DNSA reagent was pipetted into a test tube containing 1 mL of each extract (MeOH and EtOH) (1 mg/mL) and held at 95°C for 5 min. After cooling, 8 mL of distilled water was added to the solution and the absorbance was measured at 540 nm using a microplate reader (BioTek Epoch 2 Microplate Spectrophotometer). The reducing sugar content was calculated from the standard D‐glucose calibration curve (1 mg/mL), and the results were expressed as mg D‐glucose equivalent (GE) per gram dry extract weight (mg GE/g dried extract).

### 2.7 High‐Performance Liquid Chromatography With Refractive Index Detection Sugar Profiling

Soluble sugar composition was analyzed using our Shimadzu Prominence LC‐20A system with refractive index detection. Chromatographic separation was achieved on an Inertsil NH₂ column (250 × 4.6 mm, 5 μm) maintained at 35°C. The mobile phase consisted of acetonitrile: water (85:15, v/v) delivered at 1.0 mL/min. Samples (20 μL) were injected in duplicate, and quantification was performed using external calibration curves (R^2^ > 0.995) for six sugar standards (glucose, fructose, sucrose, maltose, turanose, and melibiose).

### 2.8 Protein Extraction and Quantification

Protein extraction was performed through alkaline hydrolysis, where 100 mg of dried stamen powder was digested in 5 mL 1 N NaOH at 100°C for 2 h. The cooled hydrolysate was filtered through Whatman No. 1 paper, and the filtrate was used for subsequent analysis.

For protein determination, we implemented a standardized Bradford assay (Bradford 1976), reagent formulation consisted of 100 mg Coomassie Brilliant Blue G‐250 dissolved in 50 mL 95% ethanol, followed by the addition of 100 mL 85% phosphoric acid and dilution to 1 L. The assay was performed by mixing 100 μL sample with 4 mL reagent, with absorbance measured at 595 nm after 10 min incubation. Protein concentration was calculated against a BSA standard curve (0–1 mg/mL) and expressed as BSA equivalents per gram dry weight (mg BSAE/g).

### 2.9 Carotenoid Quantification

Carotenoid extraction and quantification were performed following an optimized protocol based on (Sass‐Kiss et al. 2005). Approximately 20 mL of hexane‐acetone‐ethanol (2:1:1) solvent was added to 2 g of the dried and ground stamen sample. The solution was stirred in the dark for 30 min. The hexane phase was recovered and the lower phase was extracted a second time using 10 mL of hexane. The two fractions obtained were combined, and the absorbances were measured at 420 nm using a microplate reader (BioTek Epoch 2 Microplate Spectrophotometer). Carotenoid concentrations were calculated using the calibration curve for β‐carotene (0–100 μg/mL), and expressed as milligram equivalents of β‐carotene per 100 g of dry matter (mg β‐carotene equivalents/100 g DM) with each sample analyzed in triplicate.

### 2.10 Lycopene Quantification

Lycopene content was assessed using the (Rodriguez‐Amaya 2001) method, with three replicates. Exactly 0.1 g of sample was homogenized in 10 mL of hexane: acetone: ethanol (50:50:1, v/v/v), shaken for 10 min, and centrifuged (5000 × g, 15 min, 4°C); the supernatant was diluted 1:10 in hexane for spectrophotometric analysis. Absorbance measurements at 472 nm were converted to lycopene concentration using the molar extinction coefficient (*ε* = 3450 M^−1^ cm^−1^ in hexane) and the formula (1):

(1) $$\text{Lycopene}\;\left(\frac{\mathrm{mg}}{100\;g\;\mathrm{MS}}\;\right)=\frac{\left(\mathrm{abs}472\times \mathrm{Df}\times 1000000\times V\right)}{\left(3450\times 100\times W\right)}$$

where Df = dilution factor, *V* = extraction solvent volume, 3450 = molar extinction coefficient of lycopene in hexane, *W* = sample weight (g).

### 2.11 Condensed Tannin Quantification

Condensed tannins were quantified using an optimized vanillin‐HCl assay adapted from (Julkunen‐Tiitto 1985). 50 μL aliquots of extracts (10 mg/mL in methanol) were reacted with 1.5 mL freshly prepared 4% (w/v) vanillin solution in methanol and 750 μL concentrated HCl. After vortex mixing, samples were incubated in light‐protected vials at 25°C ± 1°C for exactly 20 min. Absorbance measurements were taken at 500 nm using a microplate reader (BioTek Epoch 2 Microplate Spectrophotometer). Quantification employed a catechin calibration curve (1 mg/mL), with results expressed as catechin equivalents (μg CE/mg extract).

### 2.12 Phenolic Compounds Analysis of *Crocus sativus* Stamens Extracts and Fractions by High‐Performance Liquid Chromatography Coupled With Diode Array Detection

Phenolic compounds in *Crocus sativus* stamen extracts and fractions were analyzed using a modified method by (Tokul‐Ölmez et al. 2020) with a Shimadzu HPLC system equipped with an LC‐20AT pump. Separation was achieved on a reverse‐phase C18 column (5 μm, 4.6 mm × 250 mm) with a column temperature of 35°C. The mobile phases consisted of 0.1% acetic acid in water (A) and 0.1% acetic acid in methanol (B), with a gradient elution program ranging from 2% to 100% B. The flow rate was set at 1.0 mL/min, and 20 μL of each sample was injected. A diode array detector (DAD) monitored phenolic compounds at a wavelength of 254 nm. Compounds were identified by comparing retention times and UV spectra with those of reference standards. Calibration curves were constructed for standard compounds, and results were expressed as mg/g of extract.

### 2.13 Gas Chromatography–Mass Spectrometry Analysis of the Petroleum Ether Fraction

#### 2.13.1 Preparation of Fatty Acid Methyl Esters

To identify the fatty acids, present in the petroleum ether fraction of *Crocus sativus* stamens, an esterification step was carried out prior to gas chromatography–mass spectrometry (GC–MS) analysis. A sample of 0.6 g of the fraction was refluxed with 16 mL of methanol and 320 μL of sulfuric acid at 50°C for 2 h under constant stirring. After the reaction, the mixture was transferred to a separatory funnel and extracted with 16 mL of chloroform and 32 mL of distilled water. The organic phase was isolated and washed twice with water, following the protocol of (Wotto et al. 2015).

### 2.14 Gas Chromatography–Mass Spectrometry Conditions and Compound Identification

The petroleum ether fraction was analyzed by gas GC–MS using a Shimadzu QP2010 system (Kyoto, Japan). A capillary column BPX‐25 (30 m × 0.25 mm i.d., film thickness 0.25 μm) was used for separation. The carrier gas was helium, with a flow rate of 3 mL/min. The oven temperature was programmed as follows: initial temperature 50°C (held for 5 min), then increased by 10°C/min to 250°C, and maintained at this temperature for 10 min. The injector temperature was set at 225°C, and the interface temperature at 250°C. Mass spectra were recorded using an electron ionization (EI) mode at 70 eV, with a scan range of m/z 40–600 and a scan speed of 5 scans/s. The ion source temperature was set at 200°C. Component identification was carried out by comparing the obtained mass spectra with the NIST 147 library database (National Institute for Standard Technology, 198 compounds; LabSolutions software version 2.5, USA). The relative abundance of each compound was calculated as the percentage of its peak area relative to the total ion current (TIC) obtained in the chromatogram.

### 2.15 In Vitro Antioxidant Activity Evaluation of *Crocus sativus* Stamens Extracts and Fractions

#### 2.15.1 DPPH Radical Scavenging Assay

The antioxidant activity of the various extracts and fractions was assessed using the DPPH radical scavenging method, as described by (Sánchez‐Moreno et al. 1998) with slight modifications. In brief, 50 μL of each sample at different concentrations (25, 50, 100, and 200 μg/mL) was mixed with 1950 μL of a freshly prepared methanolic DPPH solution (4 mg/100 mL). The mixtures were shaken and incubated in the dark at room temperature for 30 min. Absorbance was recorded at 517 nm using a microplate reader (BioTek Epoch 2 Microplate Spectrophotometer). A blank was prepared using methanol instead of the sample. Ascorbic acid (0–200 μg/mL) was used as a positive control. A decrease in absorbance reflected a higher radical scavenging ability.

IC_50_ values, corresponding to the concentration required to inhibit 50% of DPPH radicals, were determined by plotting the percentage of inhibition against concentration and applying an exponential regression model.

#### 2.15.2 ABTS Radical Scavenging Assay

Antioxidant capacity was also measured using the ABTS radical cation decolorization assay (TEAC), as described by (Miller and Rice‐Evans 1996). ABTS•^+^ was generated by reacting 20 mM ABTS with 70 mM potassium persulfate in the dark for 24 h at room temperature. The solution was then diluted in phosphate‐buffered saline (PBS, pH 7.4) to obtain an absorbance of 0.700 ± 0.020 at 734 nm. For each extract (25, 50, 100, and 200 μg/mL), 10 μL was mixed with 2.0 mL of diluted ABTS^•+^ solution. Absorbance was measured at 734 nm using a microplate reader (BioTek Epoch 2 Microplate Spectrophotometer). Solvent blanks were used for correction. TEAC values were calculated by comparing the absorbance decrease of samples to that of Trolox standards (0–200 μg/mL).

#### 2.15.3 *β*‐Carotene–Linoleic Acid Bleaching Assay

The *β*‐carotene bleaching assay was performed as an in vitro model of lipid peroxidation induced by linoleic acid‐derived radicals, following the method of (Tepe et al. 2006) with slight modifications. In this system, peroxidation of linoleic acid generates free radicals that attack and bleach *β*‐carotene, leading to a decrease in absorbance at 490 nm. Antioxidants present in the samples inhibit this bleaching, indicating lipid peroxidation suppression.

Briefly, 2 mg of *β*‐carotene was dissolved in 1 mL of chloroform. After solvent evaporation, 20 mg of linoleic acid, 200 mg of Tween 80, and 100 mL of distilled water were added under vigorous stirring to form an emulsion. Then, 50 μL of each sample (25–200 μg/mL) was mixed with 2450 μL of the *β*‐carotene/linoleic acid emulsion. Butylated hydroxytoluene (BHT, 1 mg/mL) was used as a positive control. Absorbance was measured at 490 nm using a microplate reader (BioTek Epoch 2 Microplate Spectrophotometer) immediately after mixing (*A*_0_) and after 2 h of incubation at 50°C (*A*_f_). The percentage of β‐carotene oxidation was calculated using the following formula (2):

(2) $$\%\text{Oxidation}=\left[\left(A₀-A_{f}\right)/A₀\right]\times 100$$

### 2.16 Metal Chelating Activity

#### 2.16.1 Ferrous Ion (Fe^2+^) Chelation

The Fe^2+^ chelating activity was measured according to the method by (Carter 1971), with slight modifications. Briefly, 250 μL of the sample or standard solution at various concentrations was mixed with 1 mL of acetate buffer (0.1 M, pH 4.9) and 25 μL of FeCl₂ solution (2 mM). After incubation at room temperature for 30 min, the reaction was initiated by adding 100 μL of ferrozine solution (5 mM). The mixture was further incubated for another 30 min at room temperature to allow the formation of the ferrozine–Fe^2+^ complex, which exhibits a maximum absorbance at 562 nm.

A negative control was prepared by replacing the sample with an equal volume of distilled water. The absorbance was measured at 562 nm using a 96‐well microplate reader (BioTek Epoch 2 Microplate Spectrophotometer). Ethylenediaminetetraacetic acid (EDTA) was used as a positive control (standard chelator), with concentrations ranging from 0 to 200 μg/mL. The chelating activity was expressed as the percentage inhibition of the Fe^2+^–ferrozine complex formation, calculated using the following formula (3):

(3) $$\%\text{Inhibition}=\left[\left(A_{0}-A_{1}\right)/A_{0}\right]\times 100$$

where *A*₀ is the absorbance of the control and *A*₁ that of the sample.

#### 2.16.2 Cupric Ion (Cu^2+^) Chelation

The copper ion chelating capacity was determined using the method by (Saiga et al. 2003), with slight modifications. Each 0.25 mL sample was mixed with 1 mL of 50 mM sodium acetate buffer (pH 6.0) and 25 μL of 5 mM CuSO₄. After 30 min, 25 μL of pyrocatechol violet (PV) solution was added. The mixture was incubated for an additional 30 min, and the absorbance was measured at 632 nm using a 96‐well microplate reader. EDTA (0–200 μg/mL) was used as a positive control, and distilled water served as the blank. Chelating activity was calculated using the same formula as above.

### 2.17 Statistical Analysis

Data are presented as mean ± standard error of the mean (SEM). Statistical comparisons were performed using one‐way analysis of variance (ANOVA) followed by Tukey's post hoc test, using GraphPad Prism version 8 (GraphPad Software, San Diego, CA, USA). Differences were considered statistically significant at *p* < 0.05.

## 3 Results

### 3.1 Extraction Yields

The extraction yields of the different solvent extracts and fractions are presented in Table 1. The hydromethanolic and hydroethanolic extracts showed the highest yields, approximately 49.8% and 49.25%, respectively. In contrast, the AEH exhibited the lowest yield (4.56%), while the petroleum ether fraction reached 12.05%.

**TABLE 1:** Extraction yield and tannin content of *Crocus sativus* stamen extracts and fractions.

Extract/Fraction	Yield (% dry weight)	Tannin content (μg eq catechin/g)
MeOH	49.8 ± 1.1	7.15 ± 0.28^b^
EtOH	49.25 ± 1.43	13.58 ± 0.38^a^
Petroleum ether fraction	12.05 ± 0.05	Nd
AENH	8.28 ± 0.66	7.36 ± 0.16^b^
AEH	4.56 ± 0.35	3.03 ± 0.10^d^

*Note:* Extraction yields are expressed as a percentage of the dry weight of *Crocus sativus* stamens. Values are presented as mean ± SD (*n* = 3). Different superscript letters (a–f) within the same column indicate significant differences between samples (*p* < 0.05) according to one‐way ANOVA followed by Tukey's post hoc test.

Abbreviations: AEH, hydrolyzed ethyl acetate fraction; AENH, non hydrolyzed ethyl acetate fraction; AqH, hydrolyzed aqueous fraction; AqNH, non hydrolyzed aqueous fraction; EtOH, hydroethanolic extract; MeOH, hydromethanolic extract; nd, not detected.

### 3.2 Qualitative Elemental Composition by Energy‐Dispersive X‐Ray Fluorescence Spectroscopy

The elemental composition of *Crocus sativus* stamens was qualitatively assessed using energy‐dispersive X‐ray fluorescence spectroscopy (EDXRF‐7000). Spectral analyses were conducted in two energy ranges: 0–40 keV (for heavier elements, Al–U) and 0–20 keV (for lighter elements, Na–Sc).

**FIGURE 2:**
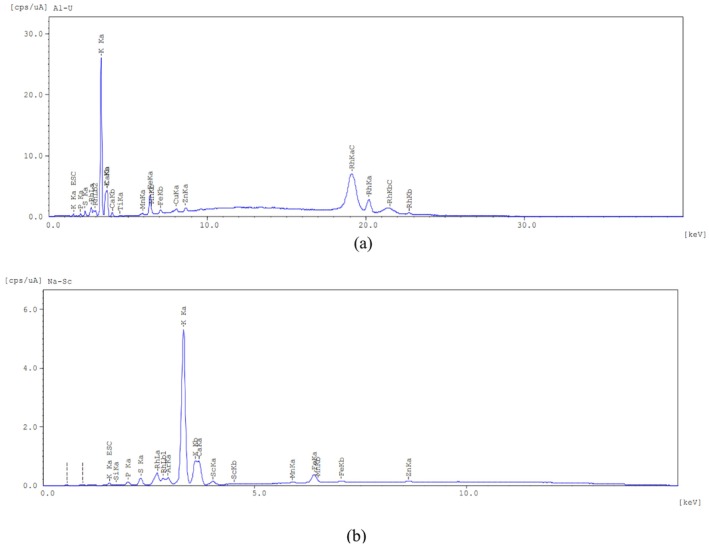
Energy‐dispersive X‐ray fluorescence (EDXRF) spectra of Crocus sativus stamens. (a) Full energy range (0–40 keV), showing major peaks corresponding to potassium (K, 3.32 keV) and calcium (Ca, 3.68 keV), along with minor signals from phosphorus (P, 2.02 keV), sulfur (S, 2.32 keV), iron (Fe, 6.40 keV), manganese (Mn, 5.92 keV), and zinc (Zn, 8.62 keV). (b) Expanded energy range (0–20 keV), highlighting additional elements including silicon (Si, 1.73 keV) and scandium (Sc, 4.02 keV), as well as low‐intensity peaks of P, S, Mn, and Fe.

In the 0–40 keV range, the most intense emission peaks corresponded to potassium (K, 3.32 keV) and calcium (Ca, 3.68 keV). Additional peaks were observed for phosphorus (P, 2.02 keV), sulfur (S, 2.32 keV), and iron (Fe, 6.40 keV). Trace elements such as manganese (Mn, 5.92 keV) and zinc (Zn, 8.62 keV) were also detected (Figure 2, a).

In the 0–20 keV range, peaks for potassium and calcium remained predominant. Additional emissions were recorded for silicon (Si, 1.73 keV), scandium (Sc, 4.02 keV), phosphorus, and sulfur. These results confirm the presence of a diverse elemental profile in the stamens, including essential macro‐ and micronutrients (Figure 2, b).

### 3.3 Quantitative Elemental Analysis by Inductively Coupled Plasma–Optical Emission Spectroscopy

Inductively coupled plasma–optical emission spectrometry (ICP‐OES) provided quantitative data on the mineral composition of *Crocus sativus* stamens. As shown in Table 1, potassium was the dominant macronutrient (3.55%), followed by phosphorus (0.52%), calcium (0.44%), and magnesium (0.31%). Regarding micronutrients, iron was detected at a notably high concentration (526.58 mg/L), followed by manganese (41.58 mg/L), zinc (25.23 mg/L), boron (12.48 mg/L), and copper (10.59 mg/L). These results highlight the nutritional potential of *Crocus sativus* stamens as a source of essential minerals. To ensure the accuracy and reliability of the ICP‐OES method, certified reference material NIST‐CRM 1203 (drinking water) was analyzed in parallel. As presented in Table 2, the experimental values closely matched the certified values, with recovery rates ranging from 99.37% to 102.63%. These results confirm the validity and precision of the analytical method used in this study.

**TABLE 2:** Mineral content in *Crocus sativus* stamens and validation of ICP‐OES analysis using NIST‐CRM 1203 (drinking water).

Macroelements				
Element	Content	Certified and experimental values of studied metals in NIST‐CRM 1203 drinking water (mg/kg)^a^		
		Certified value (mg/kg)	Experimental value (mg/kg)^b^	Recovery value (%)
Phosphorus (%)	0.52		—	—
Potassium (%)	3.55		—	—
Calcium (%)	0.44	99.78 ± 0.50	100.42 ± 0.95	100.06
Magnesium (%)	0.31	99.77 ± 0.50	100.68 ± 1.02	100.12

Microelements				
Element	Content (mg/L)	Certified value (mg/kg)	Experimental value (mg/kg)^b^	Recovery value (%)
Iron (mg/L)	526.58	200.3 ± 1.0	199.89 ± 2.05	99.89
Copper (mg/L)	10.59	200.0 ± 10	202.9 ± 0.12	101.54
Manganese (mg/L)	41.58	50.17 ± 0.25	50.02 ± 0.75	99.37
Zinc (mg/L)	25.23	1000 ± 5	1003.1 ± 7.8	102.63
Boron (mg/L)	12.48		—	—

*Note:* Values are expressed as mean ± SD (*n* = 3).

^a^ Ten times dilution of Certified NIST‐CRM 1203 Drinking Water.

^b^ Average of triplicate measurements of certified material (*p* < 0.05).

### 3.4 Total and Reducing Sugars Content in *Crocus sativus* Stamens

Total sugar content was quantified using the phenol–sulfuric acid method. The hydromethanolic extract contained 32.83 ± 1.31 g of glucose equivalents per 100 g dried extract, while the hydroethanolic extract showed 30.04 ± 1.45 g/100 g dried extract (Table 3).

**TABLE 3:** Total sugars, reducing sugars, and protein content in *Crocus sativus* stamen.

Extract/Fraction	Total sugars (g Eq glucose/100 g dried extract)	Reducing sugars (g Eq glucose/100 g dried extract)	Proteins (mg Eq BSA/100 g DW)
MeOH	32.83 ± 1.31	18.21 ± 4.90	
EtOH	30.04 ± 1.45^ns^	19.00 ± 0.60^ns^	
Dried stamens			7.01 ± 0.15

*Note:* Values are expressed as glucose equivalents for total and reducing sugars (g/100 g dried extract, DE) and bovine serum albumin (BSA) equivalents for proteins (mg/100 g dry weight, DW). Data are presented as mean ± SD (*n* = 3).

Abbreviations: EtOH, hydroethanolic extract; MeOH, hydromethanolic extract; ns, not significant compared to the hydromethanolic extract.

Reducing sugars, determined by the DNSA method, were present at 18.21 ± 4.90 g/100 g dried extract (MeOH) and 19.00 ± 0.60 g/100 g dried extract (EtOH), showing no statistically significant difference between the two solvents (Table 3).

### 3.5 Soluble Sugars Composition in *Crocus sativus* Stamens by HPLC‐RI

The HPLC‐RI analysis of soluble sugars revealed that sucrose was the predominant sugar in both MeOH and EtOH extracts. Fructose was slightly more abundant in EtOH (12.84 μg/mg extract) than in MeOH (10.23 μg/mg). Glucose levels were nearly identical in both extracts (≈4.8 μg/mg), while turanose was detected in higher amounts in EtOH (6.83 μg/mg) compared to MeOH (2.65 μg/mg). Maltose and melibiose were not detected (Table 4).

**TABLE 4:** Soluble sugar content (μg/mg) in extracts determined by HPLC‐RI.

Sugar	MeOH (μg/mg dried extract)	EtOH (μg/mg dried extract)
Fructose	10.23	12.84
Glucose	4.80	4.81
Sucrose	19.14	23.84
Turanose	2.65	6.83
Maltose	nd	nd
Melibiose	nd	nd

*Note:* Concentrations are expressed in micrograms of sugar per milligram of dried extract (μg/mg). HPLC‐RI: high‐performance liquid chromatography with refractive index detector.

Abbreviations: EtOH, hydroethanolic extract; MeOH, hydromethanolic extract; not detected.

### 3.6 Protein Content in *Crocus sativus* Stamens

The protein content in the stamens of *Crocus sativus* was evaluated using the Bradford method, with the regression equation derived from the BSA calibration curve (*y* = 1.4136*x* −0.0013, *R* ^2^ = 0.9925). The results show that the stamens contain 7.01 ± 0.15 mg of protein per 100 g of dry matter (DM) (Table 3).

### 3.7 Condensed Tanins, Carotenoids, and Lycopene

The quantification of condensed tannins using the vanillin–HCl method showed that the EtOH contained significantly higher levels (13.58 ± 0.50 μg catechin equivalents/mg extract) compared to the MeOH (10.72 ± 0.25 μg CE/mg) (*p* < 0.05) (Table 1).

Carotenoid content, expressed as β‐carotene equivalents, was higher in the EtOH extract (166.14 ± 3.10 mg/100 g dry weight) than in the MeOH extract (158.36 ± 4.12 mg/100 g DW). Lycopene levels were low in both extracts, with slightly higher content in EtOH (0.58 ± 0.01 mg/100 g) than in MeOH (0.53 ± 0.02 mg/100 g DW) (Table 5).

**TABLE 5:** Content of carotenoids and lycopene.

Carotenoids (mg Eq *β*C/100 g DW)	Lycopene (mg/100 g DW)
166.14 ± 4.83	0.58 ± 0.05

*Note:* Carotenoids: expressed as *β*‐carotene equivalents (βC), per 100 g of dry weight. Values are presented as mean ± SD (*n* = 3).

### 3.8 High‐Performance Liquid Chromatography Coupled With Diode Array Detection Results

The HPLC‐DAD analysis of *Crocus sativus* stamens performed at 254 nm identified several major compounds with varying concentrations depending on the extracts and fractions (Table 6). *p*‐Hydroxybenzoic acid was predominantly found in the hydrolyzed ethyl acetate fraction (8.30 mg/g) and the nonhydrolyzed ethyl acetate fraction (5.89 mg/g), while lower amounts were present in the hydroethanolic (0.13 mg/g), and hydromethanolic (0.38 mg/g) extracts. Epicatechin was exclusively detected in the AEH fraction at a concentration of 6.26 mg/g. Ferulic acid was also specific to the AENH fraction (1.08 mg/g). Rutin, a dominant flavonoid, reached its highest concentration in the AENH fraction (7.29 mg/g), followed by the MeOH (2.35 mg/g). Crocetin, a characteristic carotenoid pigment, was predominantly found in the EtOH extract (14.32 mg/g), MeOH (7.19 mg/g), and to a lesser extent in the nonhydrolyzed aqueous fraction (2.30 mg/g). Quercetin was present in the AEH fraction (1.94 mg/g) and in smaller amounts in the AqH fraction (1.01 mg/g). Hesperetin was primarily detected in the AENH fraction (1.15 mg/g) and absent in other extracts. Luteolin was weakly represented in the AENH fraction (0.62 mg/g) and in the MeOH extract (0.10 mg/g). Kaempferol was identified in the AENH fraction. Epicatechin was mainly identified in the AEH fraction, with a high concentration of 6.26 mg/g.

**TABLE 6:** Phenolic and flavonoid compounds in extracts and fractions determined by HPLC‐DAD (254 nm).

Compound	RT (min)	EtOH	MeOH	AqNH	AqH	AENH	AEH
*p*‐Hydroxybenzoic acid	30.867	0.13	0.38	nd	nd	5.89	8.30
Epicatechin	35.278	nd	nd	nd	nd	nd	6.26
Ferulic acid	42.564	nd	nd	nd	nd	1.08	nd
Rutin	47.527	1.61	2.35	1.5	nd	7.29	nd
Crocin	53.215	14.32	7.19	2.30	nd	nd	nd
Quercetin	55.190	nd	nd	nd	1.01	nd	1.94
Hesperetin	57.470	nd	nd	nd	nd	1.15	nd
Luteolin	57.872	nd	nd	0.10	nd	nd	nd
Kaempferol	62.458	nd	nd	nd	nd	0.62	nd

*Note:* Concentrations are expressed in milligrams per gram of dried extract (mg/g).

Abbreviations: AEH, hydrolyzed ethyl acetate fraction; AENH, non hydrolyzed ethyl acetate fraction; AqH, hydrolyzed aqueous fraction; AqNH, non hydrolyzed aqueous fraction; EtOH, hydroethanolic extract; MeOH, hydromethanolic extract; nd, not detected; RT, retention time (min).

### 3.9 Phytochemical Profiling of *Crocus sativus* Stamens by Ultra‐High Performance Liquid Chromatography Coupled With Tandem Mass Spectrometry

The ultra‐high performance liquid chromatography coupled with tandem mass spectrometry (UHPLC–MS/MS) analysis performed in negative ionization mode enabled the identification and semiquantitative evaluation of 25 phytochemicals across the different extracts and fractions of *Crocus sativus* stamens (Table 7), including phenolic acids, flavonoids (aglycones and glycosides), anthocyanins, and other polyphenolic constituents. The most abundant compound overall was chlorogenic acid, particularly concentrated in the ethyl acetate hydrolyzed fraction (AEH, 27.05%), followed by the hydromethanolic (14.60%), aqueous hydrolyzed (12.75%), and aqueous nonhydrolyzed (10.59%) extracts. Gallic acid was detected exclusively in the AEH fraction (1.57%), indicating its liberation under hydrolytic conditions. Flavonoid glycosides were predominantly found in the nonhydrolyzed extracts, with rutin exhibiting the highest content in the hydroethanolic extract (14.46%), followed by the hydromethanolic (10.68%) and ethyl acetate nonhydrolyzed (11.24%) fractions. Quercetin‐3‐*O*‐hexose‐deoxyhexose and kaempferol‐3‐*O*‐glucoside were consistently abundant in EtOH, MeOH, and AENH, while the AEH fraction was enriched in aglycones, notably quercetin (7.57%) and catechin gallate (15.59%). Delphinidin‐3‐rutinoside, the principal anthocyanin identified, reached its highest concentration in AEH (6.17%) and was present in moderate amounts in other fractions. The AqH exhibited a specific profile marked by the presence of arbutin (1.49%), absent from all other samples. The aqueous nonhydrolyzed fraction, although moderate in total phenolic content, showed a diversified profile with flavonoid glycosides (isorhamnetin‐3‐*O*‐rutinoside, 3.85%), free flavonoids (quercetin, 2.93%), and notable levels of catechin gallate (4.01%).

**TABLE 7:** Phytochemical composition (%) of *Crocus sativus* stamens extracts and fractions determined by UHPLC–MS/MS (negative ion mode).

Identification	Molecular formula	Molar mass (g/mol)	[M − H]^−^ (m/z)	EtOH	MeOH	AEH	AENH	AqH	AqNH
Gallic acid	C_7_H_6_O_5_	170.12	169.02	nd	nd	1.57	nd	nd	nd
Quercetin	C_15_H_10_O_7_	302.24	301.03	5.04	5.92	7.57	3.26	4.44	2.93
Transferulic acid	C_10_H_10_O_4_	194.18	193.05	0.33	0.55	3.86	0.91	1.39	0.73
Hesperetin	C_16_H_14_O_6_	302.28	301.07	2.61	3.32	1.96	2.36	2.75	3.89
Trimethoxyflavone	C_18_H_14_O_5_	312.29	311.08	0.31	0.35	0.27	0.53	nd	nd
Arbutin	C_12_H_16_O_7_	272.25	271.07	0.09	0.18	0.13	0.20	0.17	0.09
Apigenin	C_15_H_10_O_5_	270.24	269.04	nd	nd	nd	nd	1.49	nd
Amentoflavone	C_30_H_20_O_10_	538.46	537.12	0.01	0.02	nd	0.02	0.002	0.004
Luteolin	C_15_H_10_O_6_	286.24	285.04	0.59	0.85	0.11	0.46	0.04	1.13
Quercetin‐3‐O‐glucoside	C_21_H_20_O_12_	464.38	463.08	1.97	1.76	0.98	3.49	0.76	0.68
Quercetin‐3‐O‐glucuronic acid	C_21_H_18_O_13_	478.34	477.08	1.69	2.05	0.92	2.12	0.69	0.51
Kaempferol‐3‐O‐glucoside	C_21_H_20_O_11_	448.38	447.09	12.40	9.05	0.27	9.33	0.23	4.004
Quercetin‐3‐O‐deoxyhexosyl‐hexoside	C_27_H_30_O_16_	610.51	609.14	13.32	9.42	0.27	9.23	0.24	3.91
Isorhamnetin‐3‐O‐rutinoside	C_28_H_32_O_16_	624.54	623.16	5.66	5.65	nd	5.07	nd	3.85
Isorhamnetin‐7‐O‐pentoside	C_21_H_20_O_11_	462.39	461.08	1.01	1.04	1.46	1.56	nd	nd
Luteolin‐7‐O‐glucoside	C_21_H_20_O_11_	448.38	447.09	1.24	1.32	1.61	1.83	nd	nd
Kaempferol‐3‐O‐glucuronic acid	C_21_H_18_O_12_	478.34	477.08	0.20	0.30	0.40	0.25	0.48	0.99
Kaempferol‐3‐O‐pentoside	C_21_H1_8_O_12_	478.34	477.08	1.00	nd	0.40	0.25	0.99	0.48
Kaempferol‐3‐O‐deoxyhexosyl‐hexoside	C_27_H_30_O_15_	594.52	593.15	4.58	3.06	nd	2.10	nd	nd
Syringic acid	C_9_H_10_O_5_	198.17	197.05	0.15	0.48	nd	nd	1.11	1.05
p‐Hydroxybenzoic acid	C_7_H_6_O_3_	138.12	137.02	0.34	0.54	1.53	1.15	nd	nd
Caffeic acid	C_9_H_8_O_4_	180.16	179.03	0.87	0.94	0.87	1.28	0.72	1.93
Transcinnamic acid	C_9_H_8_O_2_	148.16	147.04	0.06	0.13	3.71	0.29	0.61	0.15
Chlorogenic acid	C_16_H_18_O_9_	354.31	353.09	10.19	14.60	27.05	13.68	12.75	10.59
Catechin/Epicatechin	C_15_H_14_O_6_	290.27	289.07	nd	nd	2.97	nd	nd	nd
Catechin gallate	C_22_H_18_O_10_	442.38	441.09	5.40	7.25	15.59	8.05	6.18	4.01
Procyanidin	C_30_H_26_O_12_	578.52	577.15	0.10	0.14	nd	0.09	nd	nd
Kaempferol	C_15_H_10_O_6_	286.24	285.04	0.69	0.97	nd	0.50	nd	0.22
Rutin	C_27_H_30_O_16_	610.52	609.15	14.46	10.68	nd	11.24	nd	4.45
Delphinidin‐3‐rutinoside	C_27_H_31_O_15_	626.53	625.15	5.45	3.86	6.17	2.77	nd	nd

*Note:* Compounds identified by molecular masses ([M − H]^−^). Contents are expressed as percentage (%) of each extract.

Abbreviations: AEH, hydrolyzed ethyl acetate fraction; AENH, non hydrolyzed ethyl acetate fraction; AqH, hydrolyzed aqueous fraction; AqNH, non hydrolyzed aqueous fraction; EtOH, hydroethanolic extract; MeOH, hydromethanolic extract; nd, not detected.

### 3.10 Fatty Acid and Volatile Compound Profiling by Gas Chromatography–Mass Spectrometry

Gas chromatography–mass spectrometry analysis of the petroleum ether fraction revealed a complex mixture dominated by fatty acid esters and hydrocarbons. A total of 11 major compounds were identified (Table 8).

**TABLE 8:** Fatty acid and lipid composition in stamens extracts by GC–MS.

No	Compound	Chemical formula	Retention time (min)	Content (%)
1	Lauric acid (Dodecanoic acid)	C_12_H_24_O_2_	18.139	4.58
2	Myristic acid (Tetradecanoic acid)	C_14_H2_8_O_2_	20.618	1.85
3	Palmitic acid (Hexadecanoic acid)	C_16_H_32_O_2_	22.861	26.52
4	Arachidic acid (Eicosanoic acid)	C_20_H_40_O_2_	23.335	4.06
5	Docosatrienoic acid (22:3 n‐3 or n‐6)	C_22_H_38_O_2_	23.892	0.30
6	Linoleic acid isomer (Octadecadienoic acid)	C_18_H_32_O_2_	24.627	26.29
7	Iso‐heptadecanoic acid (15‐Methyl‐heptadecanoic acid)	C_19_H_38_O_2_	24.705	12.20
8	Unclear identity (Octadec‐9‐enoic acid?)	C_26_H_50_O_4_	24.886	2.30
9	Oleic acid (cis‐9‐Octadecenoic acid)	C_18_H_34_O_2_	25.152	14.34
10	Stearic acid (Octadecanoic acid)	C_18_H_36_O_2_	25.335	3.06
11	Hexadecadienol (Z,Z‐8,10‐Hexadecadien‐1‐ol 6‐)	C_16_H_30_O	26.658	4.50

*Note:* Compounds were identified by comparison of retention times and mass spectra with reference standards. Retention times are in min, and contents are given in percentage (%). Major identified compounds include palmitic acid (26.52%), 8,11‐octadecadienoic acid (26.29%), and oleic acid (14.34%).

The predominant fatty acids were palmitic acid methyl ester (26.52% of total peak area), oleic acid methyl ester (14.34%), and 8,11‐octadecadienoic acid methyl ester (26.29%). Linoleic acid and stearic acid derivatives were also present in lower concentrations. Additionally, volatile hydrocarbons such as tetradecane and heptadecane were detected.

### 3.11 In Vitro Antioxidant Activities of Extracts and Fractions

The antioxidant potential of *Crocus sativus* stamens extracts and fractions was assessed using four complementary in vitro assays: DPPH, ABTS, *β*‐carotene bleaching, and metal chelation (Fe^2+^ and Cu^2+^). All samples exhibited dose‐dependent antioxidant activity, with the AEH showing the most potent effect in all tests.

### 3.12 DPPH Radical Scavenging Activity

Table 9 summarizes the DPPH radical scavenging activity of *Crocus sativus* stamens extracts. All samples exhibited dose‐dependent antioxidant activity. The AEH showed the highest activity (IC_50_ = 9.77 ± 0.57 μg/mL), comparable to ascorbic acid (IC_50_ = 19.82 ± 1.13 μg/mL), and significantly more active than the nonhydrolyzed counterpart (AENH). The hydroethanolic extract was more effective than the hydromethanolic extract, while aqueous fractions exhibited the weakest activity (IC_50_ > 140 μg/mL; *p* ≤ 0.001).

**TABLE 9:** Antioxidant and chelating activities of *Crocus sativus* stamens extracts and fraction (IC_50_, μg/mL).

Assay	IC50 (μg/mL)									
	MeOH	EtOH	AEH	AENH	AqH	AqNH	AA	Trolox	BHT	ETDA
DPPH	50.63 ± 0.21**	27.34 ± 1.23 ns	9.77 ± 0.57 ns	54.62 ± 0.72**	231.40 ± 5.78****	143.04 ± 8.79****	19.82 ± 1.13	—	—	—
ABTS	45.68 ± 0.84***	23.44 ± 0.34 ns	23.23 ± 0.15 ns	40.27 ± 0.30***	80.41 ± 4.51****	81.27 ± 2.33****	—	15.57 ± 0.08	—	—
β‐Carotene Bleaching	57.55 ± 1.73***	37.57 ± 0.60**	28.53 ± 0.92 ns	62.80 ± 4.25***	72.23 ± 1.70***	162.88 ± 8.67****	—	—	16.54 ± 0.12	—
Iron chelation	32.72 ± 4.65 ns	27.54 ± 0.07 ns	14.68 ± 0.06 ns	23.27 ± 0.80 ns	88.50 ± 14.65***	68.08 ± 8.29***	—	—	—	9.21 ± 0.11
Copper chelation	24.69 ± 0.36**	23.46 ± 0.25**	13.01 ± 0.08*	27.24 ± 0.54***	36.99 ± 2.46***	43.61 ± 1.62***	—	—	—	13.23 ± 0.10

*Note:* IC_50_ values (μg/mL) expressed as mean ± standard deviation (*n* = 3). Statistical comparisons were performed against the respective standard for each assay: ascorbic acid (AA) for DPPH, Trolox for ABTS, and butylated hydroxytoluene (BHT) for *β*‐carotene bleaching.

Abbreviations: AEH, hydrolyzed ethyl acetate fraction; AENH, nonhydrolyzed ethyl acetate fraction; AqH, hydrolyzed aqueous fraction; AqNH, nonhydrolyzed aqueous fraction; EDTA, Ethylenediaminetetraacetic acid (standard for iron and copper chelation); EtOH, hydroethanolic extract; MeOH, hydromethanolic extract; ns, not significant.

*p < 0.05; **p < 0.01; ***p < 0.001; ****p < 0.0001.

### 3.13 ABTS Radical Scavenging Assay

The antioxidant potential of *Crocus sativus* stamens extracts and fractions was assessed based on their capacity to scavenge the ABTS^+^ radical cation. IC_50_ values (Table 9) revealed that the EtOH exhibited the highest activity among crude extracts (IC_50_ = 23.44 ± 0.34 μg/mL), followed by the MeOH. Among the fractions, AEH demonstrated the strongest antioxidant activity (IC_50_ = 23.23 ± 0.15 μg/mL), comparable to Trolox (IC_50_ = 15.57 ± 0.08 μg/mL). The AENH showed moderate activity, while the aqueous fractions (AqH and AqNH) were the least active, with IC_50_ values > 80 μg/mL, significantly lower than Trolox (*p* ≤ 0.001).

### 3.14 Effect of Extracts and Fractions of *Crocus sativus* Stamens on *β*‐Carotene Bleaching

The antioxidant potential of *Crocus sativus* stamens extracts and fractions was assessed through their ability to inhibit *β*‐carotene oxidation induced by linoleic acid. The results showed a concentration‐dependent decrease in β‐carotene degradation, confirming a dose‐responsive antioxidant effect. At 200 μg/mL, the reference antioxidant BHT showed the strongest inhibition (5.6% oxidation), followed by AEH, which limited oxidation to 9.9%. IC_50_ values (Table 9) supported these findings. Among the crude extracts, the EtOH (IC_50_ = 37.57 ± 0.6 μg/mL) exhibited the highest activity (IC_50_ = 32.83 ± 2.88 μg/mL), followed by MeOH (IC_50_ = 57.55 ± 1.73 μg/mL) extracts. Among the fractions, AEH demonstrated the strongest antioxidant activity (IC_50_ = 28.53 ± 0.92 μg/mL), closely approaching BHT (IC_50_ = 16.54 ± 0.12 μg/mL), while AENH showed moderate activity (IC_50_ = 62.80 ± 4.25 μg/mL). The aqueous fractions were less active, with IC_50_ values of 72.23 ± 1.7 μg/mL (AqH) and 162.88 ± 8.67 μg/mL (AqNH), which were significantly higher than those of BHT (*p* ≤ 0.001).

### 3.15 Metal Chelating Power

#### 3.15.1 Iron Chelating Power

Table 9 illustrates the iron chelating ability of the different extracts and fractions from the stamens of *Crocus sativus* . EDTA, used as a reference, exhibited a chelation percentage of 94.10% with an IC_50_ of 9.21 ± 0.11 μg/mL. The results showed that among the extracts, the EtOH extract displayed the best chelation activity, followed by the MeOH extract. As for the fractions, the AEH fraction demonstrated the highest chelating activity at 93.58%, followed by the AENH, AqNH, and AqH fractions. Additionally, an increase in chelation activity proportional to the concentration used was observed in all extracts and fractions.

Table 9 indicates that the best IC_50_ values were obtained for the AEH fraction (14.68 ± 0.06 μg/mL) and the EtOH extract (27.54 ± 0.07 μg/mL), while the AENH fraction presented an IC_50_ of 23.27 ± 0.8 μg/mL. In contrast, the aqueous fractions (hydrolyzed and nonhydrolyzed) showed lower activity compared to EDTA, with IC_50_ values of 88.50 ± 14.65 μg/mL and 68.08 ± 8.29 μg/mL, respectively. These results highlight that the AEH fraction and the EtOH extract possess the best iron chelation capacity among the analyzed fractions and extracts.

#### 3.15.2 Copper Chelating Power

Table 9 illustrates the copper chelating activity of the different extracts and fractions from the stamens of *Crocus sativus* , assessed by the formation of the Cu^2+^‐PV complex. EDTA, used as a standard, demonstrated a chelating power of 95.84% with an IC_50_ of 13.23 ± 0.1 μg/mL. Among the tested extracts, the AEH fraction exhibited the best chelating activity, with a chelation percentage of 78.84% and an IC_50_ of 13.01 ± 0.08 μg/mL. This was followed by the EtOH extract (IC_50_ = 23.46 ± 0.25 μg/mL), MeOH extract (IC_50_ = 24.69 ± 0.36 μg/mL), AENH fraction (IC_50_ = 27.24 ± 0.54 μg/mL), The aqueous fractions (hydrolyzed and nonhydrolyzed) exhibited the weakest chelation activity compared to EDTA, with IC_50_ values of 36.99 ± 2.46 and 43.61 ± 1.62 μg/mL, respectively.

## 4 Discussion

The present study highlights *Crocus sativus* stamens as an underexploited but valuable source of bioactive compounds, essential minerals, and antioxidants, supporting their potential inclusion in nutraceutical and functional food formulations.

Elemental analysis revealed high concentrations of potassium (3.55%), phosphorus (0.52%), calcium (0.44%), and iron (526.58 mg/L), which play critical roles in cellular functions such as enzymatic catalysis, energy transfer, and redox homeostasis. These findings align with those reported by (Cardone et al. 2020), who identified potassium as the most abundant mineral in saffron by‐products, particularly in tepals and stamens followed by calcium and magnesium. Notably, the iron content found in stamens in this study is significantly higher than that reported in *Crocus sativus* tepals by (Ahmadi Shadmehri et al. 2019), suggesting stamens could represent a superior source of bioavailable iron for nutraceutical applications. Regarding carbohydrate composition, the hydromethanolic extract exhibited a total sugar content of 32.83 g/100 g, exceeding values previously reported in *Crocus sativus* tepals (25–28 g/100 g) (Moratalla‐López et al. 2019), indicating a richer carbohydrate profile in the stamens. Sucrose was the major sugar detected, consistent with earlier observations by Serrano‐Díaz, Sánchez, Alvarruiz, and Alonso (2013); Serrano‐Díaz, Sánchez, Martínez‐Tomé, et al. (2013). Additionally, the protein content (7.01 mg/100 g dry weight) is noteworthy and supports potential applications in functional foods, as previously described in saffron floral waste (Montoro et al. 2012).

In terms of phytochemical composition, the AEH was particularly enriched in bioactive aglycone phenolics, including quercetin (7.57%) and catechin gallate (15.59%), as confirmed by UHPLC–MS/MS, which was associated with superior antioxidant capacity. These findings are consistent with previous reports demonstrating that hydrolysis enhances phenolic bioactivity by releasing aglycones (Alañón et al. 2016; Mamri et al. 2025). The dominance of chlorogenic acid across all extracts reinforces their radical scavenging capacity (Liu et al. 2021).

The AEH fraction exhibited the highest antioxidant activity in multiple assays. Its DPPH scavenging capacity (IC_50_ = 9.77 μg/mL) and metal‐chelating activities were comparable or superior to standard antioxidants such as ascorbic acid and EDTA. These results agree with (Chen et al. 2020), who observed that saffron's floral parts retain high antioxidant activity due to minor phenolic compounds and possible synergistic effects. Moreover, the *β*‐carotene bleaching assay confirmed the ability of AEH to inhibit lipid peroxidation, a result consistent with the antioxidant behavior of similar ethyl acetate fractions in hawthorn (Alirezalu et al. 2020). This suggests potential applications in oxidative stress management, particularly in food preservation or cosmetic formulations. The GC–MS analysis revealed a lipid profile dominated by palmitic and oleic acid methyl esters, which are known for their roles in cell membrane integrity and anti‐inflammatory activities. These results are comparable to those obtained by (Wotto et al. 2015) in other medicinal plants, reinforcing the multifunctional value of stamen‐derived petroleum ether fractions. The enhanced antioxidant properties of the AEH fraction can be attributed not only to the presence of specific compounds but also to their synergistic interactions. This is supported by several studies demonstrating that combinations such as quercetin and catechin gallate exhibit amplified biological effects (Chen et al. 2019; Shahidi and Zhong 2015). The marked difference in activity between hydrolyzed and nonhydrolyzed fractions underlines the importance of structural forms of phenolics in determining bio‐efficacy.

To further valorize the findings, it is crucial to consider the next steps for translational application, particularly for the AEH fraction, which exhibited the most potent antioxidant activity across all assays. Although the in vitro results are promising, the bioavailability and metabolic fate of these compounds in vivo remain largely unexplored.

Several studies have highlighted that aglycones such as quercetin, epicatechin, and gallic acid identified in high amounts in the AEH fraction often display enhanced biological activities compared to their glycosylated counterparts due to better absorption and cellular uptake (Manach et al. 2005; Yang et al. 2019). However, their in vivo stability, metabolism, and potential synergistic interactions require further investigation to confirm their efficacy after oral administration. Future research should focus on pharmacokinetic studies to evaluate the absorption, distribution, metabolism, and excretion (ADME) of the AEH fraction. Additionally, preclinical in vivo models are necessary to validate its antioxidant and protective effects in biological systems, particularly in oxidative stress‐related pathologies such as metabolic syndrome, neurodegenerative diseases, or inflammation (Scalbert and Williamson 2000; Xiang et al. 2024). Such studies will help determine whether the bioactive potential observed in vitro translates into significant health benefits in vivo, and will provide valuable information for the potential development of functional ingredients or nutraceutical formulations derived from *Crocus sativus* stamens.

## 5 Conclusion

This study provides novel and compelling evidence that *Crocus sativus* stamens traditionally regarded as floral waste represent a valuable and sustainable source of bioactive molecules. Through a comprehensive chemical and functional characterization, this work revealed a remarkable richness in essential minerals, sugars, proteins, phenolic acids, flavonoids, and carotenoids. The originality of this research lies in the valorization of an underexplored plant part, offering new insights into its nutritional and antioxidant potential. Among all tested extracts and fractions, the ethyl acetate hydrolyzed fraction (AEH) exhibited the strongest antioxidant capacity across multiple in vitro assays, likely attributed to its high concentration of aglycone phenolics and synergistic interactions between compounds.

These findings not only expand the current knowledge on saffron by‐products but also open promising perspectives for the development of natural antioxidants applicable in the food, nutraceutical, and cosmetic industries. Future research should focus on elucidating the bioavailability, safety, and in vivo biological effects of these stamen‐derived compounds to further support their industrial valorization and integration into functional products.

## Author Contributions

Conceptualization, original draft writing, reviewing, and editing: Samira Mamri, Sanae Baddaoui, Dilaycan Çam, Cansel Çakir, Mohammed Roubi. Formal analysis, investigations, funding acquisition, reviewing, and editing: Raffaele Conte, Yusuf Sıcak, Mehmet Öztürk, Mohammed Choukri, Musaab Dauelbait. Resources, data validation, data curation, and supervision: Gehan M. Elossaily, Yousef A. Bin Jardan, Abdel‐Rhman Z. Gaafar, Abdeslam Asehraou, Ennouamane Saalaoui.

## Funding

This work is financially supported by the Ongoing Research Funding Program (ORF‐2025‐686), King Saud University, Riyadh, Saudi Arabia, and AlMaarefa University under project number MHIRSP2025‐018.

## Ethics Statement

The authors have nothing to report.

## Consent

The authors have nothing to report.

## Conflicts of Interest

The authors declare no conflicts of interest.

## Supporting information


**Data S1:** fsn371282‐sup‐0001‐Supinfo.pdf.

## Data Availability

All data generated or analyzed during this study is included in this published article.
