# Supplementary material for: From Floral By‐Product to Bioactive Resource: Phytochemical Profiling and Antioxidant Potential of Crocus sativus Stamens
Source: Food Sci Nutr. 2025 Dec 13;13(12):e71282. doi: 10.1002/fsn3.71282 (PMC12701521; doi:10.1002/fsn3.71282)
Supplement: Supplementary file 1 — Data S1: fsn371282‐sup‐0001‐Supinfo.pdf. [file FSN3-13-e71282-s001.pdf]

HPLC-DAD chromatograms (Supplement to Main Results)

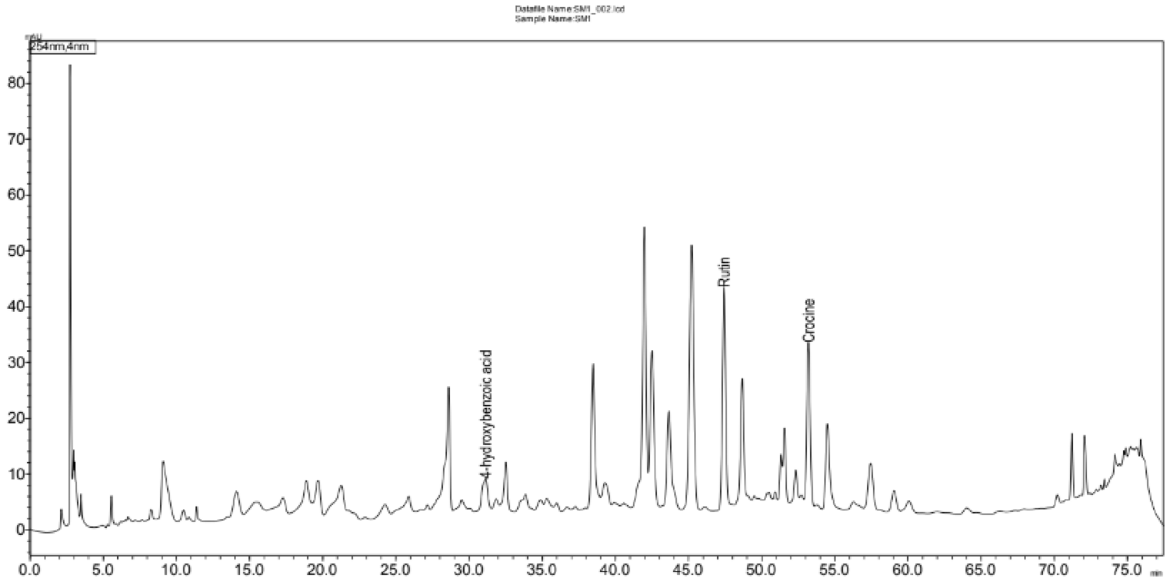

Chromatographic profile (HPLC-DAD) of the hydro-methanolic extract of Crocus sativus stamens at 254 nm, showing the peaks of the identified compounds (4-hydroxybenzoic acid, rutin, crocin).

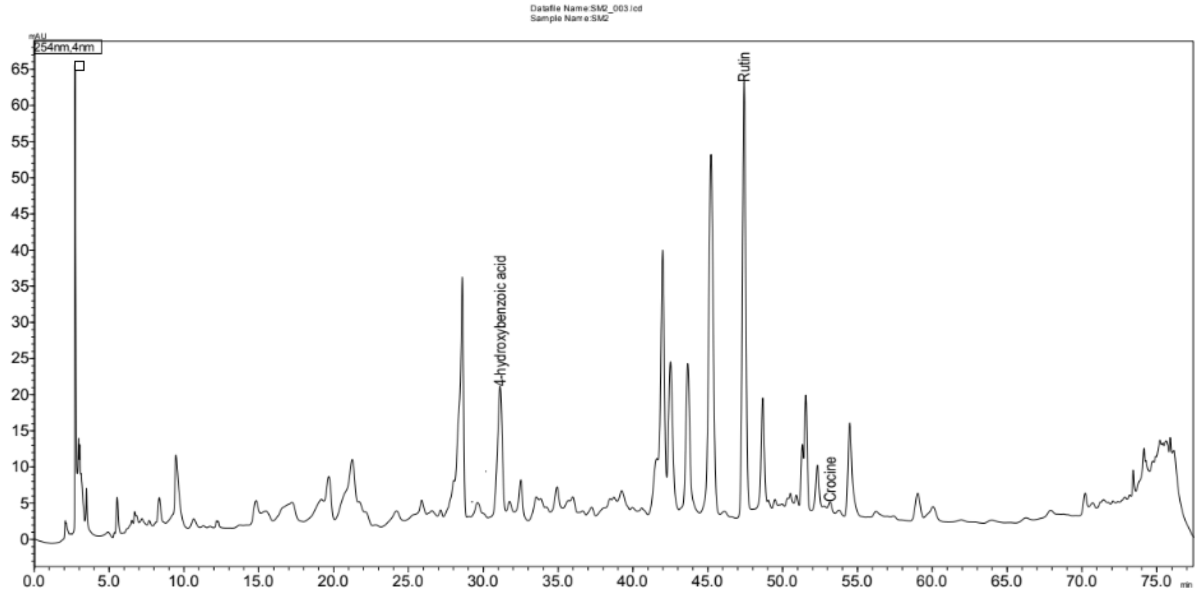

Chromatographic profile (HPLC-DAD) of the hydroethanolic extract of Crocus sativus stamens at 254 nm, showing the peaks of the identified compounds (4-hydroxybenzoic acid, rutin, crocin).

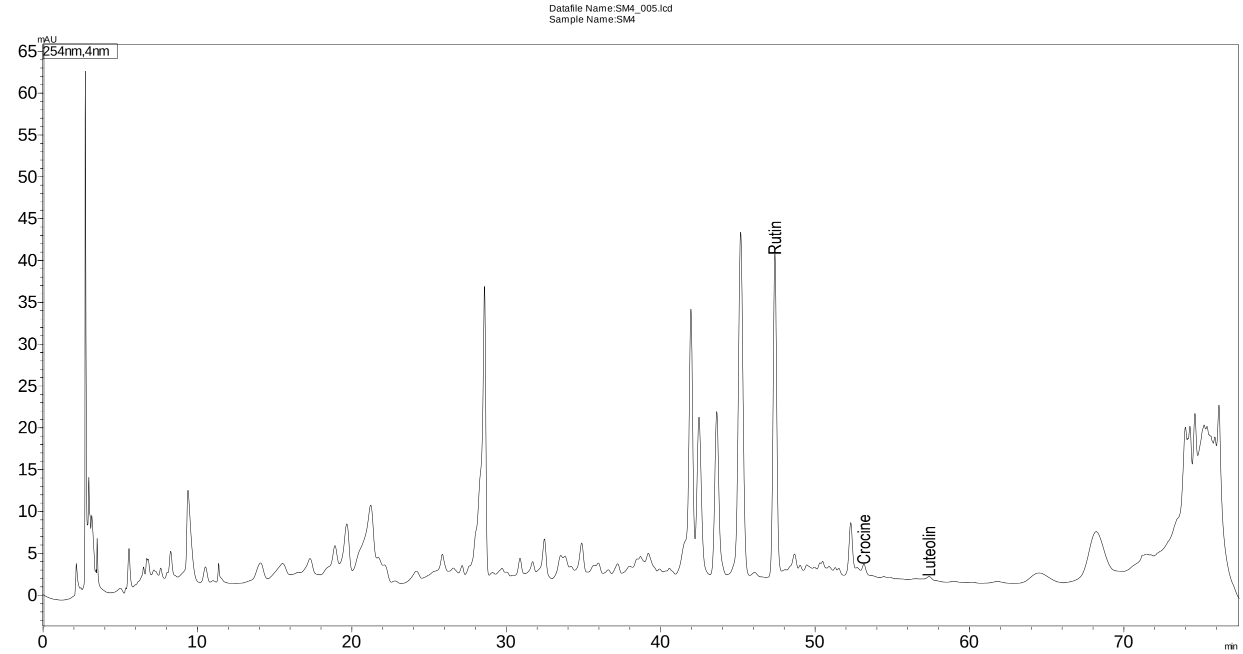

Chromatographic profile (HPLC-DAD) of the unhydrolysed aqueous fraction of Crocus sativus stamens at 254 nm, showing the peaks of the identified compounds (rutin, crocin, luteolin)

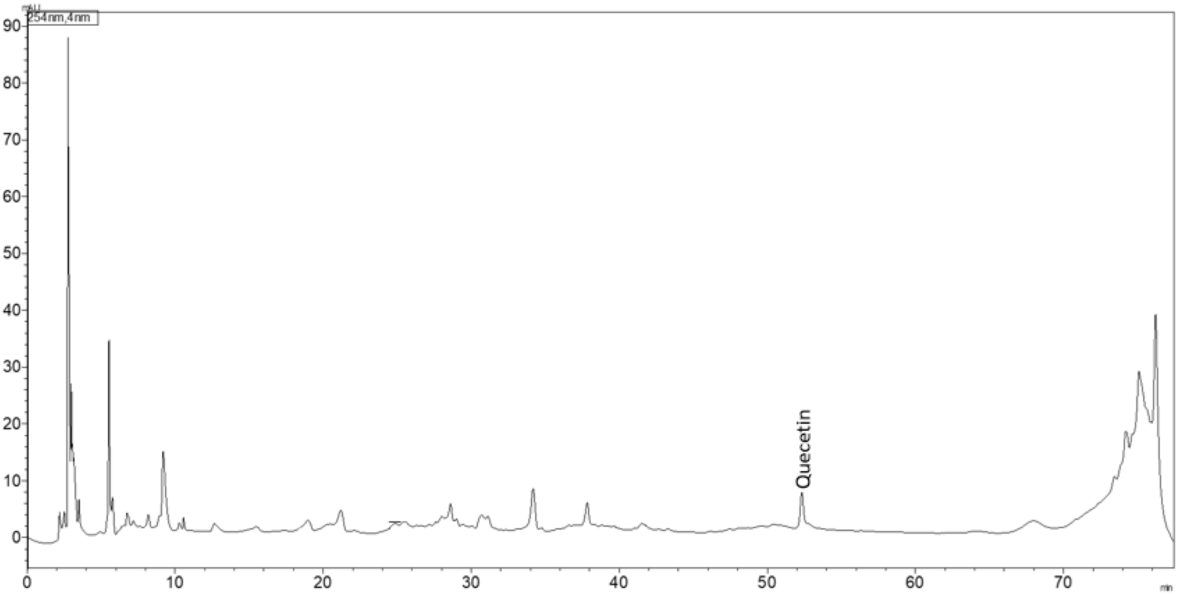

Chromatographic profile (HPLC-DAD) of the hydrolysed aqueous fraction of Crocus sativus stamens at 254 nm, showing the peaks of the identified compounds (quercetin).

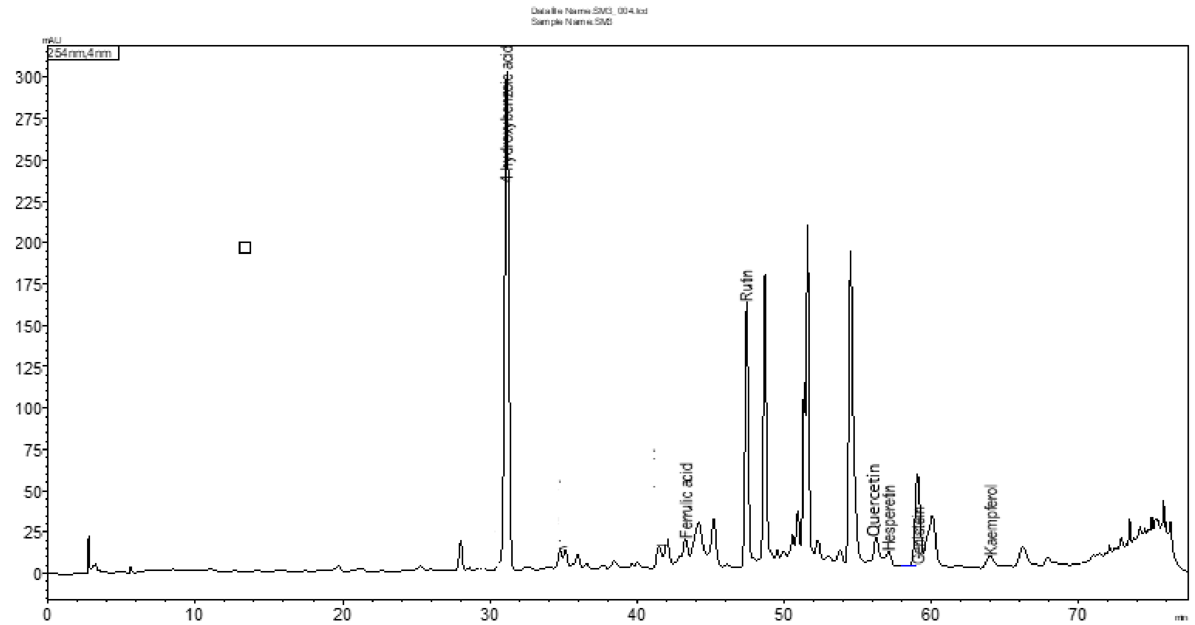

HPLC-DAD chromatogram of the unhydrolysed ethyl acetate fraction of Crocus sativus stamens at 254 nm, showing the peaks of the identified compounds (4-hydroxybenzoic acid, ferulic acid, quercetin, hesperetin, kaempferol, rutin).

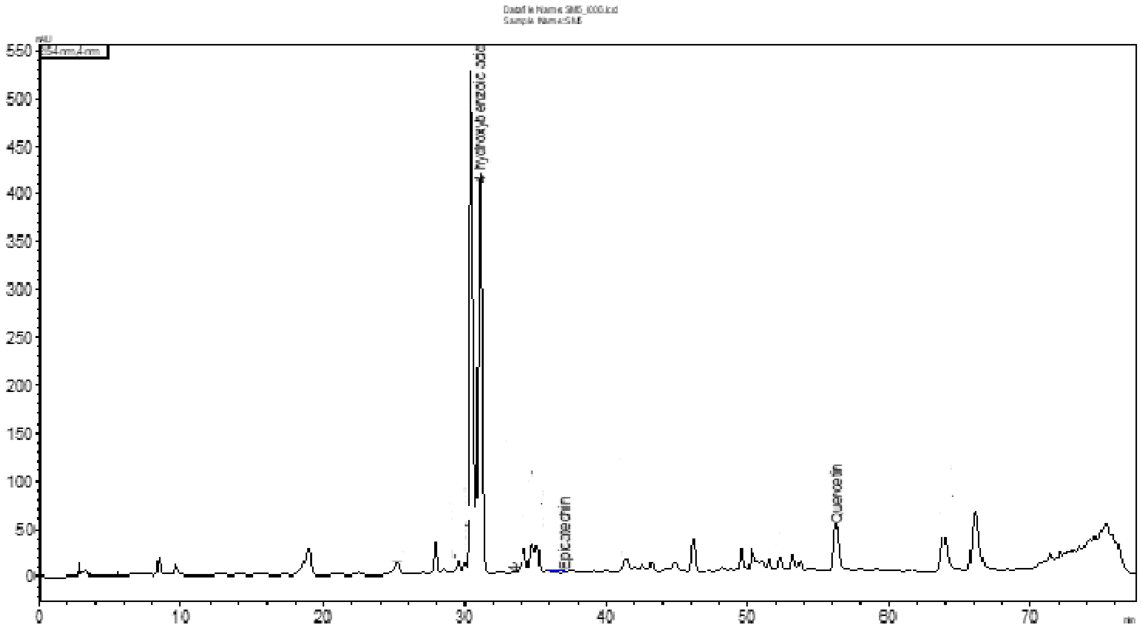

HPLC-DAD chromatogram of the unhydrolysed ethyl acetate fraction of Crocus sativus stamens at 254 nm, showing the peaks of the identified compounds (4-hydroxybenzoic acid, quercetin, epicatechin).

UHPLC-MS/MS Chromatograms (Supplement to Main Results)

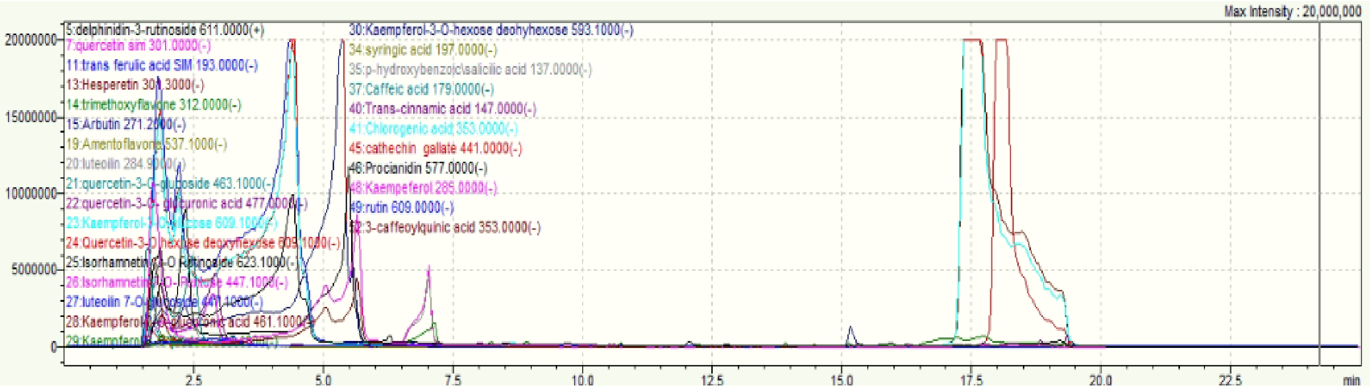

Chromatogram illustrating the main metabolites identified in the hydroethanolic extract of Crocus sativus stamens by UHPLC-MS-MS. Each peak is attributed to a specific molecule, identified by its name and molecular mass (mentioned in brackets) in negative ion mode. The colours, generated automatically by the software, are repeated due to the limited number of shades available. The precise correspondence between the molecules, their distinct chromatographic peaks and their specific retention times (expressed in minutes) is detailed in the appendix. This chromatogram highlights significant phytochemical diversity, including flavonoids, phenolic acids and anthocyanins.

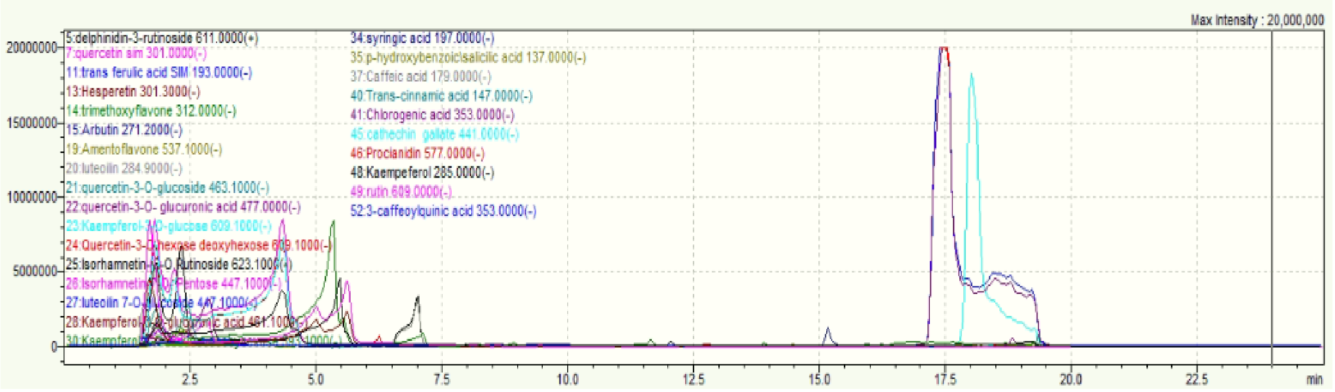

Chromatogram obtained by UHPLC-MS-MS illustrating the metabolites identified in the hydro-methanolic extract of Crocus sativus stamens. The peaks, represented in different colours automatically assigned by the software, correspond to the molecules detected, each annotated with its name and respective molecular mass. This chromatogram highlights the diversity of metabolites present in the extract, including flavonoids, phenolic acids and anthocyanins. The repetition of colours is due to the limited number of shades available in the software.

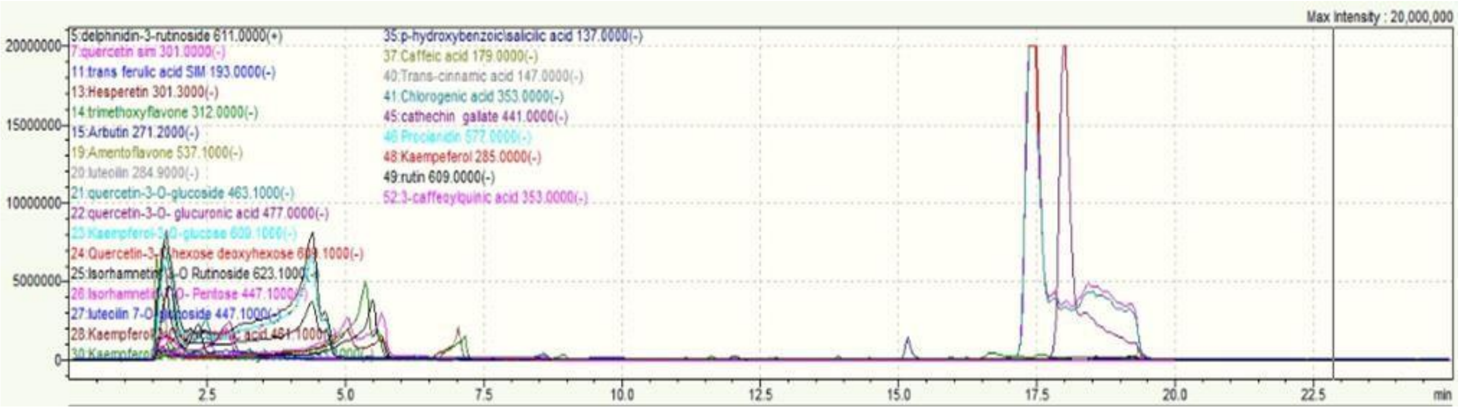

Chromatogram of the main compounds identified by UHPLC-MS/MS in the hydrolysed ethyl acetate fraction. The peaks, represented by different colours assigned automatically by the software, correspond to the molecules detected. Each peak is annotated with the name of the compound and its respective molecular mass. This chromatogram highlights the chemical composition of the fraction, comprising mainly flavonoids, phenolic acids and other bioactive metabolites. The repetition of colours is due to the limited number of shades available in the software.

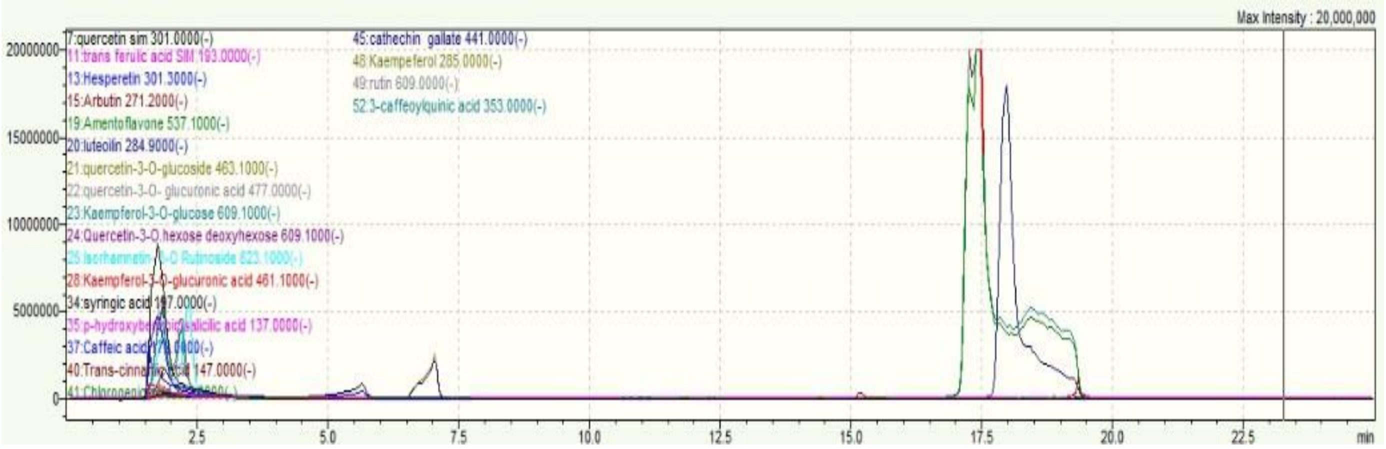

Chromatogram of the main compounds identified by UHPLC-MS-MS in the non-hydrolysed ethyl acetate fraction.

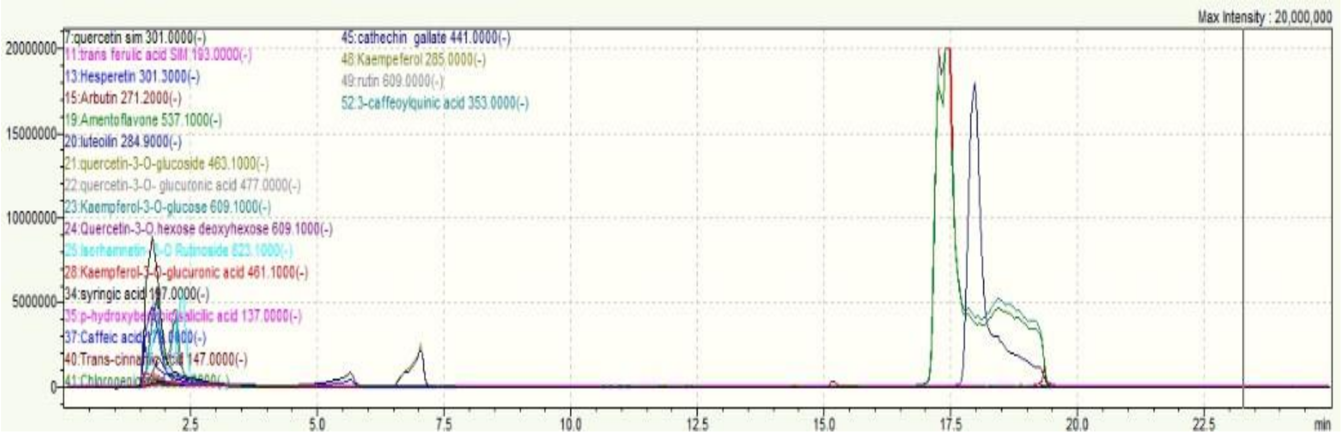

Chromatogram of the main compounds identified by UHPLC-MS-MS in the hydrolysed aqueous fraction.

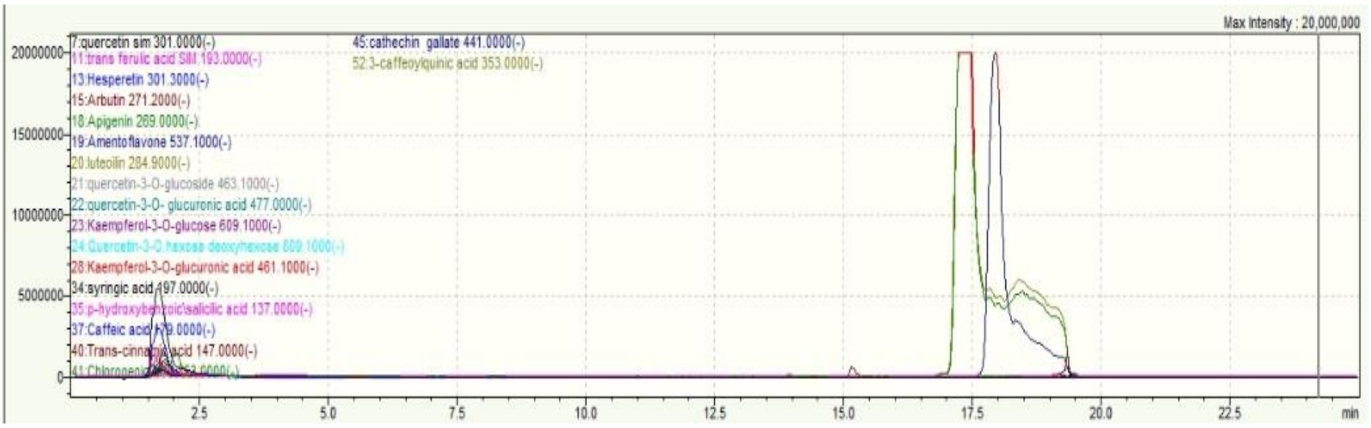

Chromatogram of the main compounds identified by UHPLC-MS-MS in the non-hydrolysed aqueous fraction.

GC-MS chromatograms (Supplement to Main Results)

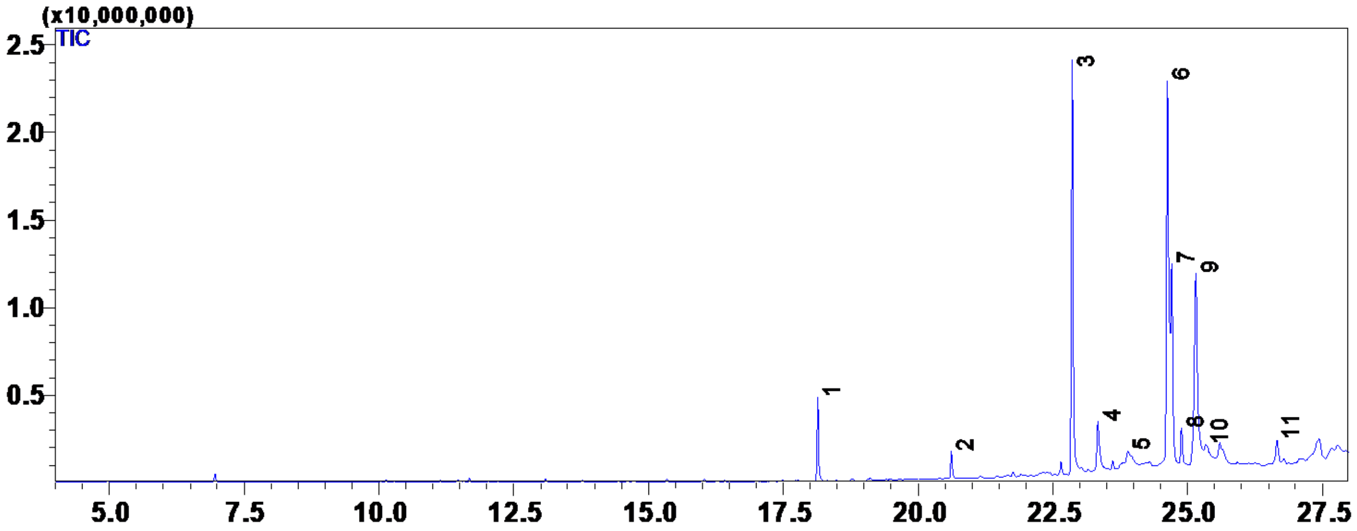

Chromatographic profile (GC-MS) of the petroleum ether fraction of Crocus sativus stamens, showing annotated peaks of identified lipid compounds. The analysis was performed by gas chromatography coupled with mass spectrometry (GC-MS). The main peaks, annotated by numbers, were identified by comparing retention times and mass spectra with reference standards. Chromatographic conditions included a capillary column (BPX 25), a temperature gradient (50 °C to 250 °C) and a carrier gas flow rate (helium at 3 ml/min). The compounds corresponding to the annotated peaks are as follows: 1 (lauric acid), 2 (tetradecanoic acid), 3 (palmitic acid), 4 (arachidic acid), 5 (8,11,14-docosatrienoic acid), 6 (8,11-octadecadienoic acid), 7 (heptadecanoic acid, 15-methyl-), 8 (octadec-9-enoic acid), 9 (oleic acid), 10 (stearic acid), and 11 (Z,Z-8,10-hexadecadien-1-ol).
